# Controlled released naringin-loaded liposome/sucrose acetate isobutyrate hybrid depot for osteogenesis *in vitro* and *in vivo*

**DOI:** 10.3389/fbioe.2022.1097178

**Published:** 2023-01-05

**Authors:** Di Meng, Jinlin Song, Yin Yi, Jihong Li, Ting Zhang, Yu Shu, Xiaohong Wu

**Affiliations:** ^1^ Stomatological Hospital of Chongqing Medical University, Chongqing, China; ^2^ Chongqing Key Laboratory of Oral Diseases and Biomedical Sciences, Chongqing, China; ^3^ Chongqing Municipal Key Laboratory of Oral Biomedical Engineering of Higher Education, Chongqing, China

**Keywords:** sucrose acetate isobutyrate, anionic liposomes, injectable, local drug delivery, controlled release

## Abstract

**Introduction:** A common problem in bone tissue engineering is that the burst release of active osteogenic factors is not beneficial for osteogenesis. This study aimed to prepare naringin (Ng) liposomes to reduce the burst release of Ng and improve new bone formation.

**Methods:** We synthesized Ng liposomes using the thin-film hydration method. Drug-encapsulation efficacy experiments were conducted using the ultracentrifugation technique. The morphology and size distributions of freezedried liposomes were determined by transmission electron microscopy and dynamic light scattering. The Ng liposomes and Ng-lipo/sucrose acetate isobutyrate (SAIB) depots were characterized using Fourier transform infrared spectroscopy and in vitro release studies. After implantation of the Ng-lipo/SAIB depots, in vitro osteoblast-liposome interactions and *in vivo* osteogenesis were tested.

**Results:** The formulation of freeze-dried Ng liposomes via an optimized recipe yielded nanosized (136.9 nm) negatively charged particles with a high encapsulation efficiency (~76.3%). Their chemical structure did not change after adding SAIB to the Ng liposomes. The burst release was reduced dramatically from 74.4% to 23.7%. *In vivo*, after 8 weeks, the new bone formation rate in the calvarial defects of Sprague-Dawley rats receiving Ng-lipo/SAIB was 57% compared with 25.18% in the control group (*p* = .0003).

**Discussion:** Our results suggested that Ng-lipo/SAIB hybrid depots could serve as candidate materials for drug delivery in bone regeneration applications.

## 1 Introduction

The oral maxillofacial bone is a hot spot for tumors, inflammation, trauma, and congenital disease. The reconstruction of oral and maxillofacial bone caused by diseases, such as fractures or bone tumor resections, is a critical challenge for dental clinicians.

Naringin (Ng), a flavanone, is an important active ingredient in Chinese traditional medicine that is widely used to treat bone defects. Previous studies revealed that Ng works not only by stimulating bone formation (Ramesh et al., 2021; Wu et al., 2021), but also by inhibiting the growth of osteoclasts or encouraging their apoptosis (Hirata et al., 2009; Li et al., 2014). In addition, Ng was reported to increase bone formation *via* inhibition of the β-Hydroxy β-methylglutaryl-CoA (HMG-CoA) reductase and activation of the *BMP2* promotor (bone morphogenetic protein 2 (Wong and Rabie, 2006).

However, Ng is highly lipophilic with poor drug solubility and low cellular uptake and, thus, cannot enter the membrane and the nucleus efficiently, which suggests that Ng had the necessity of a well-designed drug delivery system. (Zheng et al., 2022). Liposomes constitute artificial vesicles obtained from natural phospholipid bilayers, in which hydrophobic entities get loaded into the lipid bilayer (Guimarães et al., 2021). They are biocompatible and have been extensively investigated for use in a variety of industries, including food (Mozafari et al., 2008), cosmetics (Fakhravar et al., 2016), medication delivery (Michalski et al., 2019; Tiwari G. et al., 2020; Tiwari R. et al., 2020), and tissue engineering (Lee et al., 2021). Previous studies showed that liposomes significantly enhance the solubility of drugs *in vitro* (Liu et al., 2013) and improve the therapeutic efficacy of drugs by increasing interactions with cells (Zheng et al., 2022), making liposomes a potential strategy to overcome the limitations of Ng therapeutic efficacy. Nevertheless, liposome-released drugs have significant problems with burst release (Joseph et al., 2017; Hu et al., 2018; de Almeida et al., 2019). Even the sustained release effect of some liposome hybrid reservoirs is not ideal (Elkhoury et al., 2020; Mohanty et al., 2020).

Sucrose acetate isobutyrate (SAIB) is a fully esterified sucrose derivative that is generally regarded as safe by the Food and Drug Administration (FDA) (Jølck et al., 2014). Many studies have reported that SAIB is an efficient material to control the burst release of drugs from sustained release systems (Lin et al., 2012; Lin et al., 2015). In our previous studies, we successfully designed an SAIB-based hybrid depot that significantly reduced initial drug release from 66.18% to 2.92% (Zhang et al., 2021) and from 63.20% to .00% (Zhang et al., 2020). Thus, in this study, we first prepared Ng liposomes using the film hydration method and characterized their properties, including their drug-encapsulation efficacy, morphology, size distribution, and drug release. Then, these Ng liposomes were incorporated into a SAIB depot to better control Ng release. *In vivo* experiments were then conducted to evaluate the bone regeneration potential of the fabricated Ng-lipo/SAIB hybrid depots.

## 2 Materials and methods

### 2.1 Materials and animals

Commercial soybean lecithin (98% purity) and cholesterol (99% purity) were both purchased from Macklin Biochemical Co., Ltd. (Shanghai, China), and Ng was obtained from Sigma-Aldrich (St. Louis, MO, United States). SAIB was supplied by Yuanye Bio-Technology Co., Ltd. (S25342, Shanghai, China), and chloroform and methanol were provided by Concord Technology Co., Ltd. (Tianjin, China). Sucrose was obtained from Sigma-Aldrich. Tween 80 (MBQ1046, Mengbio, Chongqing, China) was used for drug release experiments. Alpha-modified Eagle’s medium ( α-MEM, HyClone, Logan, UT, United States), fetal bovine serum (FBS, Gibco, Melbourne, Australia), antibiotics (Sigma-Aldrich), phosphate-buffered saline (PBS, Gibco), and trypsin-EDTA (Beyotime, Jiangsu, China) were used in the cell culture of osteoblasts. All chemicals used were analytically pure. Sprague-Dawley rats were obtained from the Institute of Experimental Animal Center of Chongqing Medical University. Rats were fed a standard laboratory diet with a 12 h/12 h light/dark cycle under specific pathogen-free conditions and had at least 1 week of acclimatization before any animal experiment. All experimental procedures were approved by the Ethics Committee of the Affiliated Stomatological Hospital of Chongqing Medical University.

### 2.2 Preparation of freeze-dried Ng liposomes

Ng Liposomes were prepared using the film hydration method, as previously described by Bangham et al. (Bangham et al., 1965) by mixing different ratios of lipid/cholesterol (100 mol% of lipids, 80:20 mol%, 70:30 mol%, 60:40 mol%, and 50:50 mol%) (Briuglia et al., 2015) and Ng at a drug-to-lipid ratio of 1:20 (mol/mol). The mixture was dissolved in chloroform/methanol (2:1, v/v) and then dried to a thin lipid film under a vacuum using a rotatory evaporator (RE-2000A, Shanghai Yarong Biochemical Instrument Factory, Shanghai, China). The liposomes were kept in the flask overnight under a vacuum for the total removal of residual solvents. The dry lipid film was rehydrated with PBS (8:1 sucrose: lipids, mass ratio) (Guimarães et al., 2019) to obtain a lipid suspension with a final Ng concertation of .38 mg/ml.The resulting product was extruded through a .2 μm membrane 6 times using a hand-held mini extruder to obtain the liposome with the desirable size. Finally, the Ng liposome solution was freeze-dried for 48 h at −80°C in a freeze-dryer and then stored in a −20°C refrigerator until use.

### 2.3 Characterization of freeze-dried Ng liposomes

#### 2.3.1 Drug-encapsulation efficacy (EE) and drug-loading efficiency (LE)

According to previous studies, the drug EE and LE experiments were conducted using the ultracentrifugation technique (Li et al., 2018; Lin and Qi, 2021). EE was calculated as a percentage of the amount of drug inside the liposomes (encapsulated drug) compared with the total amount of drug (encapsulated and non-encapsulated drug). Briefly, 1 mg of freeze-dried Ng liposomes, which contains .14 mg Ng, was dissolved in 1.0 ml of PBS and blended completely for 5 min to obtain a sample solution. The sample solutions were centrifuged at 12,000 rpm for 10 min (Centrifuge 5418R; Eppendorf, Hamburg, Germany), and the supernatants were carefully extracted and analyzed at a wavelength of 282 nm (Elkhoury et al., 2020) using a ultra violet-visible (UV-Vis) spectrophotometer (ND-2000; Thermo Fisher Scientific, Waltham, MA, United States). The concentration of free Ng was calculated from the calibration curve. Similarly, the LE represented the total amount of Ng in the liposome sample, which was obtained by dissolving 1 mg of freeze-dried Ng liposomes in 1.0 ml of methanol and sonicating for 5 min until completely dissolved, followed by centrifugation at 12,000 rpm for 10 min. The supernatant was measured as described above. All experiments were carried out six times. The EE and LE of Ng were calculated using equations A and B.

Equation A:

$$EE=1-\frac{the\;weight\;of\;free\;Ng}{the\;weight\;of\;encapsulated\;and\;non-encapsulated\;Ng}\times 100\%$$

Equation B:

$$LE=\frac{\;the\;weight\;of\;Ng\;in\;the\;liposomes}{the\;weight\;of\;liposome\;sample\;}\times 100\%$$

#### 2.3.2 Particle size, polydispersity index (PDI), and zeta potential analysis

The freeze-dried Ng liposomes were dissolved in PBS for analysis. Before use, the samples were obtained by extruding the solution through a .22 μm microporous membrane three times at 25°C. The particle size distribution of the sample was determined using dynamic light scattering (DLS) *via* a particle size analyzer (NanoBrook 90plus PALS; Brookhaven Instruments Corporation, Holtsville, NY, United States) at 25°C, and the intensity was detected at 90°. The PDI and zeta potential of the freeze-dried Ng liposomes were determined using the same instrument. All measurements were averaged using at least three replicates, and Brookhaven software was used for the analysis.

#### 2.3.3 Transmission electron microscopy (TEM)

The morphology of the freeze-dried Ng liposomes was visualized using TEM (JEOL JEM-1400Plus, Tokyo, Japan). The liposomal solution was diluted with PBS before being placed on a copper grid. 2% phosphotungstic acid was added to stain the formulation. After drying at room temperature, the prepared thin film was observed using TEM.

### 2.4 Preparation of Ng/SAIB and freeze-dried Ng liposomes/SAIB (Ng-lipo/SAIB) depots

3.8 mg of Ng was dissolved in SAIB to obtain Ng/SAIB depots. Similarly, several freeze-dried Ng liposomes (equal to 3.8 mg Ng) were dispersed into SAIB by stirring at 55°C for 5 min to prepare Ng-lipo/SAIB depots. The final loading of the Ng/SAIB and Ng-lipo/SAIB depots preparation was 3.8 mg/ml.

### 2.5 Fourier transform infrared spectroscopy (FTIR)

FTIR (Thermo Fisher Scientific Nicolet iS5) was used to qualitatively analyze the Ng, Ng liposomes, and Ng-lipo/SAIB hybrid depots. All samples were observed using attenuated total reflection in the spectral range 500–4,000 cm^−1^.

### 2.6 *In vitro* release

Freeze-dried Ng liposomes and free Ng (both equal to 3.8 mg Ng) were transferred to dialysis bags (Mw cutoff = 3,500 Da, 25 mm × 1 m, Yibo Biotechnology Co., Ltd., Shanghai, China) (Wang et al., 2018). The dialysis bags were immersed in a centrifuge tube containing 50 ml of PBS solution (pH 7.4, 2% tween 80 (v/v)). Then, 1 ml of the Ng-lipo/SAIB depots and Ng/SAIB depots, respectively, were injected into 20 ml of the previous release buffer in a cylindrical glass bottle (22*58 mm). All the samples were incubated in a ZWY-110X30 reciprocal shaking water bath (Zhicheng Inc., Guangzhou, China) at 37°C. At each predetermined time point, 1 ml of the release buffer was removed and replaced with 1 ml of fresh medium. The 1 ml samples were analyzed using a UV-Vis spectrophotometer. All experiments were carried out in triplicate. The drug release rate was calculated using equation C.

Equation C:

$$E=\frac{V_{E}\sum_{1}^{n-1}C_{i}+V_{0}C_{n}}{M_{0}}\times 100\%$$

E is the drug release rate at each collection time point and V0, VE, and M0 are constants (1 ml, 1 ml, and 3.8 mg, respectively) in this study. The indices i and n are the numbers of experimental time points. Ci and Cn are the drug concentrations at the i and n time points, respectively.

### 2.7 Osteoblast/SAIB-based depot interactions

#### 2.7.1 Cell culture

Sprague-Dawley rats (2–3 days old) were euthanized with CO_2_, and their cranial bones were collected after 75% alcohol immersion disinfection. The bones were cut into pieces and cultured for several days to obtain osteoblast cells. The osteoblast cells were cultured in α-MEM with 10% FBS and 100 U/ml antibiotics (penicillin-streptomycin-amphotericin) at 37°C in 5% CO_2_ and the culture medium was replaced every second day.

#### 2.7.2 Cytotoxicity of the drug delivery systems toward osteoblastic cells

The cytotoxicity of the Ng-lipo/SAIB depots was assessed using Annexin V-fluorescein isothiocyanate (FITC)/propidium iodide (PI) (Yeasen Biotechnology Co., Ltd. Shanghai, China) flow cytometry and a Cell Counting Kit 8 (CCK-8) assay. The concentration of the drug was based on our previous experiments (Yang et al., 2019). 3 ml of Ng/SAIB depots and Ng-lipo/SAIB depots were immersed in 50 ml of basal culture medium (containing α-MEM with 10% FBS and 100 U/mL antibiotics), respectively, and then incubated at 37°C for 24 h to obtain their extracts. Next, the extracts were sterilized through a .22 μm filter for subsequent experiments.

For flow cytometry, the osteoblast cells were seeded in a 6-well plate. After 24 h of cell adhesion, the old medium was removed, and 3 ml of Ng-lipo/SAIB extracts per well was added to treat the cells. After incubation at 37°C for 24 h, 10^6^ cells were collected in cold PBS and resuspended in 100 μL of 1 × binding buffer. An aliquot of 100 μl cells was then incubated with 5 μL FITC Annexin V and 10 μL of PI. After incubation at 25°C for 15 min in the dark, 400 μl of 1 × binding buffer was added to each sample. Flow cytometry analysis was performed using a flow cytometry apparatus (Influx, BD, Franklin Lakes, NJ, United States).

For the CCK-8 assay, the osteoblast cells were seeded at a density of 5 × 10^3^ cells/well in a 96-well plate. After the cells were cultured in basal medium for 1 day, the old medium was discarded, and 100 μl of the Ng/SAIB and Ng-lipo/SAIB extracts were added to treat the cells, respectively. Meanwhile, 100 μl of the basal culture medium was used as the control. After 24 h, the cell culture medium was replaced with 100 μl of fresh culture medium and 10 μl of CCK-8. After 3 h, the optical density (OD) values were measured at 450 nm using an enzyme-linked immunosorbent assay (ELISA) plate reader (Bio-Tek, Winooski, VT, United States).

#### 2.7.3 Cell differentiation assessed by alkaline phosphatase (ALP) levels

The three different groups (blank, Ng/SAIB, Ng-lipo/SAIB-treated) of osteoblast cells were seeded at a density of 2 × 10^4^ cells per well in a 24-well plate, and the basal culture medium was then changed to an osteogenic-inducing medium. After 3 and 5 days of incubation, the level of ALP in the cells was measured using a commercial ALP colorimetric assay kit (Beyotime, Shanghai, China). The OD values were measured at 410 nm using an ELISA plate reader. After 7 days, the cells were stained using a 5-bromo-4-chloro-3-indolyl phosphate (BCIP)/nitro blue tetrazolium (NBT) Alkaline Phosphatase Color Development Kit (Beyotime).

### 2.8 Animal experiments

#### 2.8.1 Generation of a calvarial bone defect animal model

Adult male Sprague-Dawley rats (8–10 weeks old, *n* = 18) were randomly divided into the following three groups (*n* = 6/group): 1) Control (empty defect), 2) Ng/SAIB depots, and 3) Ng-lipo/SAIB depots. After being anesthetized by the spontaneous inhalation of isoflurane (2% isoflurane in 100% oxygen, 1 L/min), a 5 mm diameter full-thickness defect was produced at one side of the calvarium under low-speed drilling and standard saline irrigation. The incisions were stitched up after the defects were implanted with either the Ng/SAIB depots (.1 ml, .38 mg Ng) or Ng-lipo/SAIB depots (.1 ml, .38 mg Ng). After surgery, 3 days of intraperitoneal injections of penicillin (1 mg/kg) were given to prevent infection.

#### 2.8.2 Micro-computed tomography (micro-CT)

At 2- and 8-weeks after surgery, rats were sacrificed by anesthesia overdose. The harvested skulls were fixed in 10% neutral buffered paraformaldehyde and analyzed using a micro-CT (Viva CT40, SCANCO Medical, Brüttisellen, Switzerland) at a resolution of 17.5 mm using the energy of 70 kV and 114 mA. The defect region was identified using a cylindrical contour. The new bone formation rate (BV/TV %) was then calculated within this fixed volume of interest using SCANCO analysis software.

#### 2.8.3 Histological and histomorphometric analyses

After micro-CT imaging, the entire skulls were decalcified using 10% ethylenediaminetetraacetic acid (EDTA) solution. Serial sections with 5 mm thickness were prepared along the coronal plane and stained using hematoxylin and eosin (HE) (Servicebio, Wuhan, China) and Masson trichrome (Servicebio). Three central sections were randomly selected along the coronal plane for further histomorphometric measurements.

#### 2.8.4 Immunohistochemistry (IHC)

The expression of the early osteogenic marker RUNX family transcription factor 2 (Runx-2) was detected at 2 weeks. The expression of the late osteogenic marker osteocalcin (OCN) was evaluated at 8 weeks. Image analysis was performed using ImagePro Plus 6.0 (Media Cybernetics, Rockville, MD, United States)

#### 2.8.5 Statistical analyses

Data are presented as the mean ± standard deviation (SD). One- or two-way analysis of variance (ANOVA) was used for data analysis. Post hoc analysis was conducted using Tukey’s multiple comparisons test. All statistical analyses were performed using GraphPad Prism version 7 (GraphPad Software, San Diego, C\A, United States). A *p*-value of .05 or less in a two-tailed test was considered statistically significant.

## 3 Results

### 3.1 Characterization of freeze-dried Ng liposomes and Ng-lipo/SAIB depot

Compared with the other formulations, the 70:30 (lipid/cholesterol) formulation presented the highest Ng content, with a mean EE of 76.3% (±2.8%), although some of the differences were not significant (Table 1). Meanwhile, the mean LE of this formulation was 13.5% (±1.2%). According to the above data, we chose a formulation of 70:30 (lipid/cholesterol) at a drug-to-lipid ratio of 20:1 for further experiments.

**TABLE 1:** Encapsulation efficacy of freeze-dried Ng liposomes with different cholesterol concentrations. (*n* = 6).

Formulation (lipid/cholesterol, mol/mol)	Encapsulation efficacy (EE) ± SD, (%)	Drug-loading efficiency (LE) ± SD, (%)
100:0	70.1 ± 1.5	12.5 ± 0.9
80:20	71.9 ± 1.8	12.2 ± 1.1
70:30	76.3 ± 2.8	13.5 ± 1.2
60:40	74.8 ± 3.6	12.6 ± 0.6
50:50	69.3 ± 1.1	12.3 ± 0.9

**FIGURE 1:**
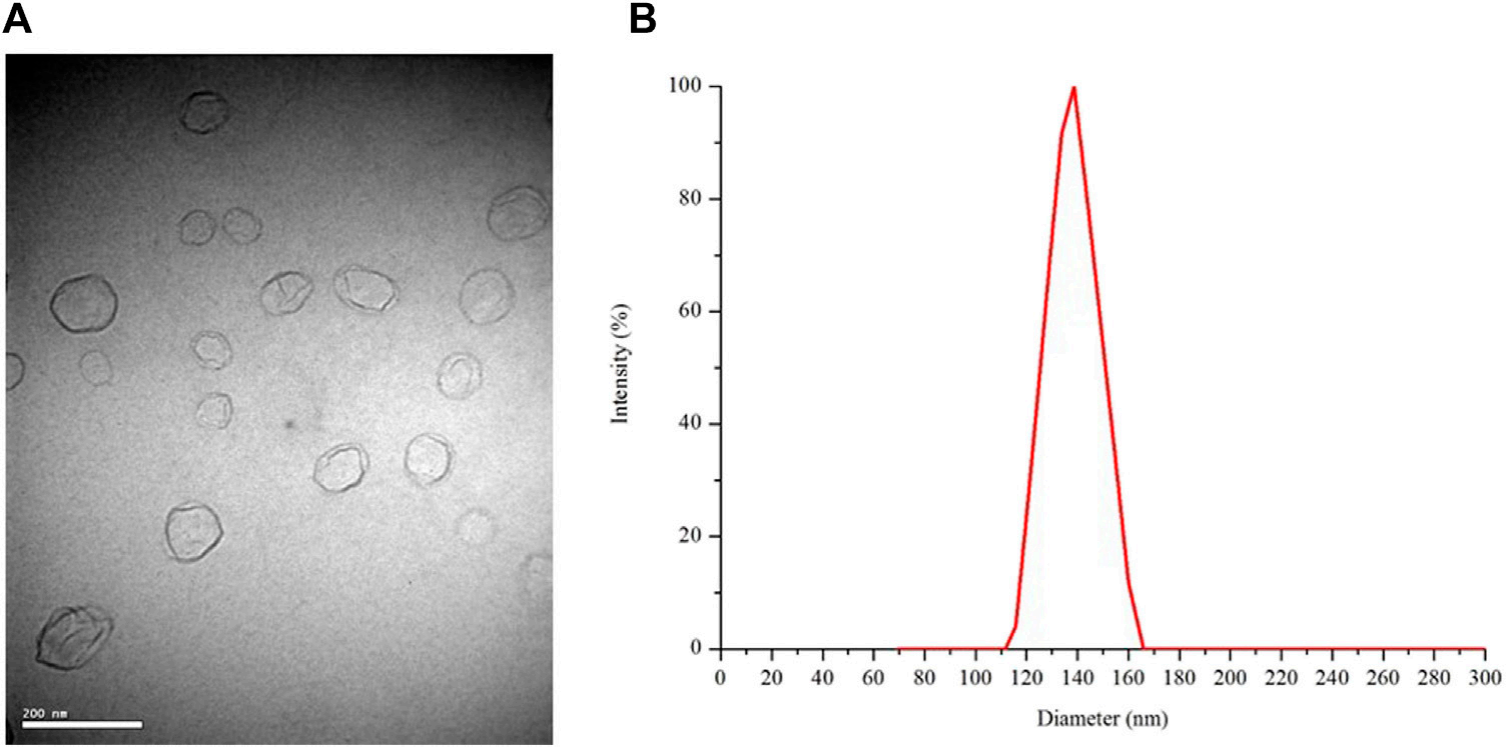
TEM image at 10,000 times magnification **(A)** and the size distribution of freeze-dried Ng liposomes **(B)**.

The morphology of the freeze-dried Ng liposomes was observed by TEM. As shown in Figure 1A, the liposomes appeared as spherical nanoparticles with a relatively homogeneous size, and no aggregation or fusion was observed. The mean diameter of the freeze-dried Ng liposomes was 136.9 nm (±16.4 nm), which was consistent with the results obtained from the TEM image (Figure 1B). The PDI was .139 ± .012. The negative zeta potential was −59.2 ± 6.2 mV.

**FIGURE 2:**
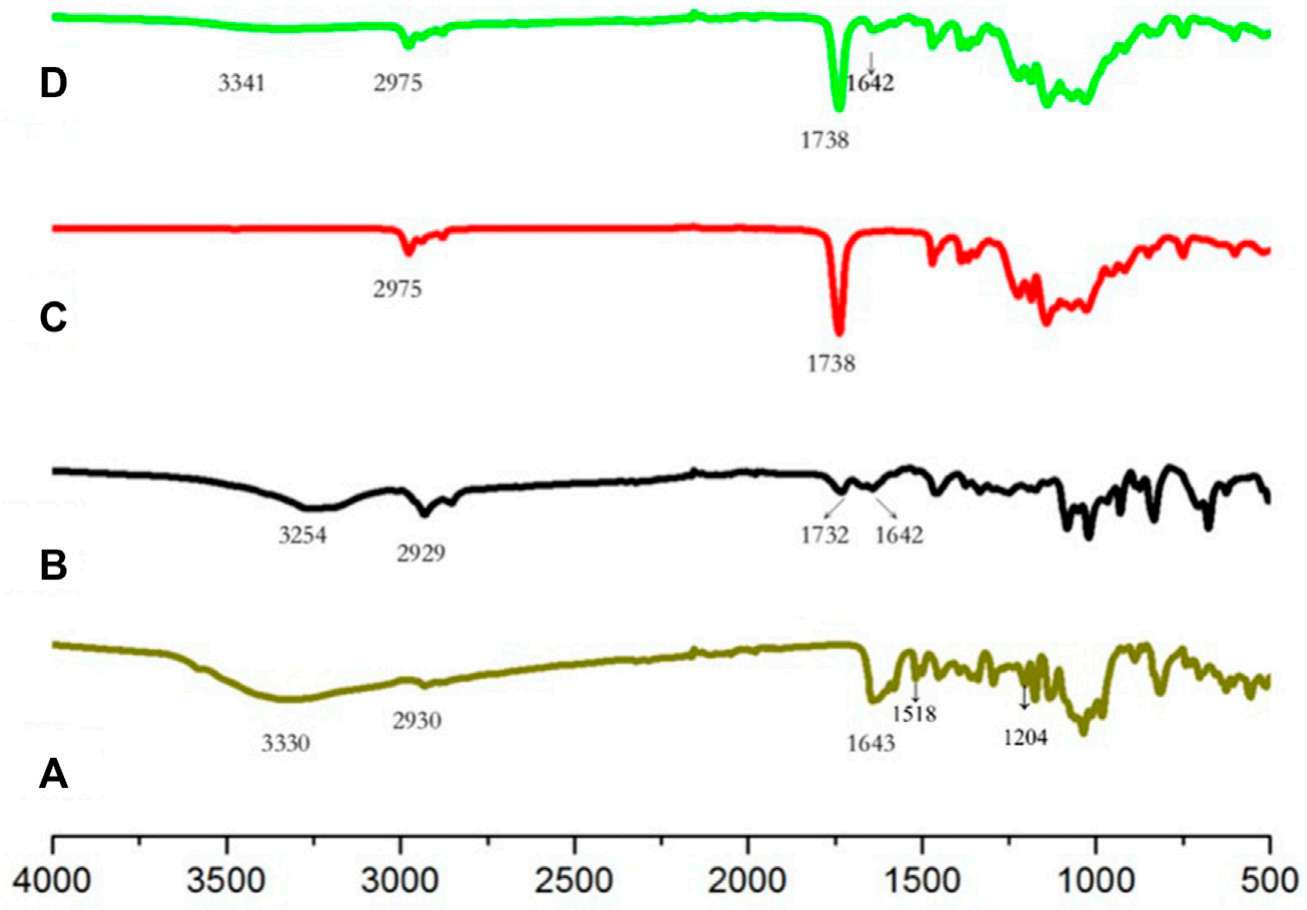
FTIR spectra of Naringin **(A)**, Ng liposomes **(B)**, SAIB **(C)**. and Ng-lipo/SAIB **(D)** depots.

Figure 2 shows the FTIR spectra of the Ng, Ng liposomes, SAIB, and Ng-lipo/SAIB depots. Accordingly, the FTIR spectrum of Ng exhibited characteristic bands at 1643 cm^−1^ and 3330 cm^−1^ (Wang et al., 2018). The other characteristic bands of Ng included the aromatic at 1518 cm^−1^ and OH phenolic at 1,204 cm^−1^ (Wang et al., 2022). In the Ng liposomes, the characteristic bands all weakened, shifting towards lower wavenumbers. The decrement of the drug bands in the liposomes indicated drug encapsulation. SAIB showed the peaks of the–CH3 at 2,975 cm^−1^ and of the–C=O at 1738 cm^−1^. These peaks could be plainly seen in the Ng-lipo/SAIB depot spectra without any significant shifting.

### 3.2 *In vitro* release of Ng liposomes and SAIB-based depots

**FIGURE 3:**
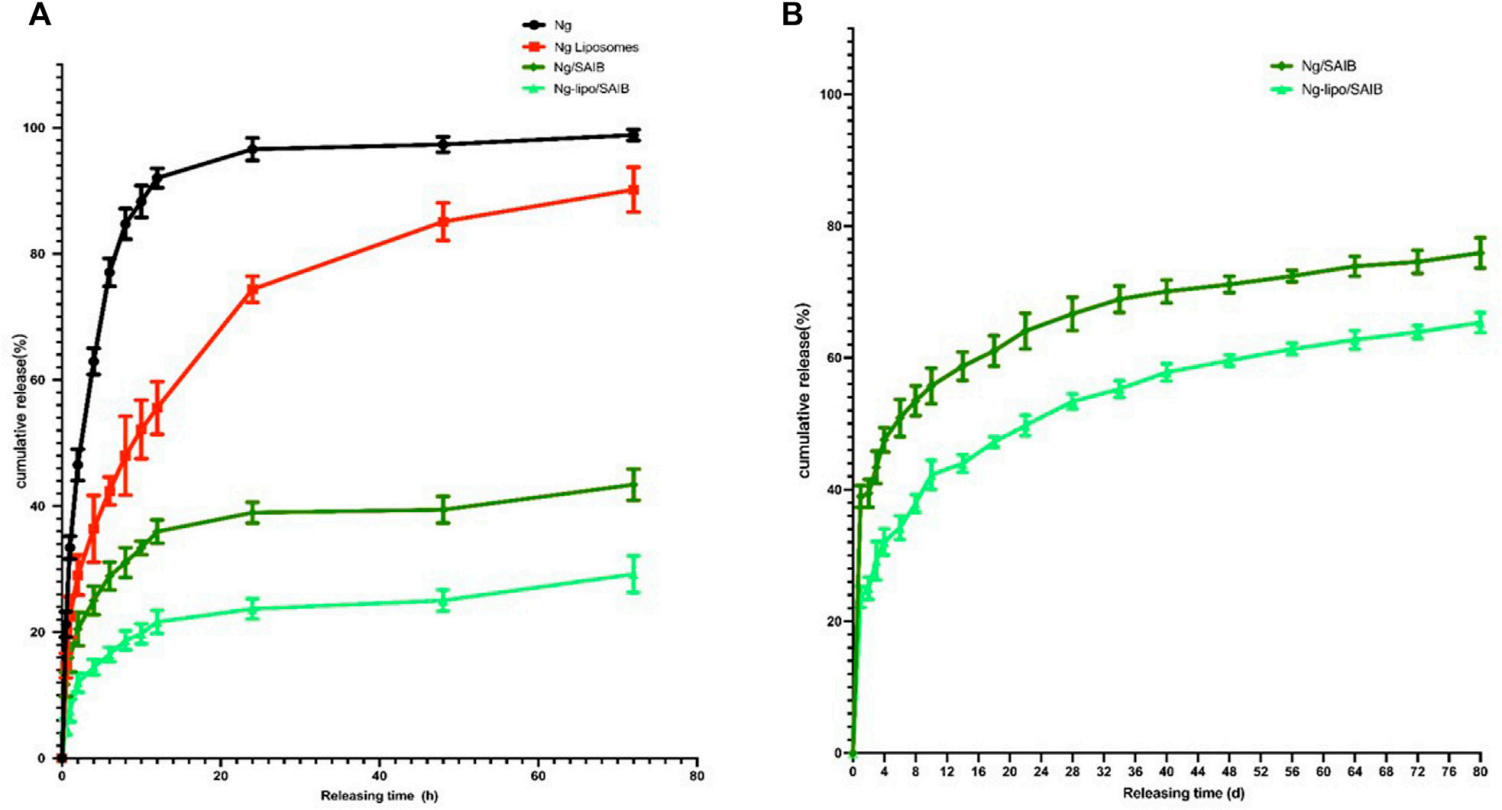
*In vitro* releases of Ng, Ng liposomes, Ng/SAIB, and Ng-lipo/SAIB during 1–3 days **(A)** and Ng/SAIB, Ng-lipo/SAIB during 3–80 days **(B)** from depots at 37°C. (*n* = 3).

The *in vitro* release profiles of Ng, Ng liposomes, Ng-SAIB, and Ng-lipo/SAIB depots over 1–3 days and Ng-SAIB and Ng-lipo/SAIB depots over 3–80 days are shown in Figure 3. Ng took about 10 h to be completely released from the dialysis bag into the medium. The release patterns of the Ng liposomes were characterized by an initial burst. For example, 74.4% of the drug was released from Ng liposomes after 24 h, and 90.2% after 72 h, while in the Ng-lipo/SAIB depots, only 23.7% was released in 24 h and 29.2% in 72 h. Figure 3B summarizes the drug release profiles for the prepared SAIB-based depots up to 80 days and over time. The release of the drug was continuous and gentle. Table 2 presents the best fit obtained with the Ritger–Peppas equation (Ritger and Peppas, 1987), which showed the best correlation with the data for Ng-lipo/SAIB release (R^2^ > .995).

**TABLE 2:** Evaluation of drug release kinetics of SAIB-based depots according to the Ritger-Peppas equation.

Ng liposomes	Ng/SAIB	Ng-lipo/SAIB
M_t_ = 0.22 × (t^0.36^)	M_t_ = 0.21 × (t^0.17^)	M_t_ = 0.11 × (t^0.25^)
*R* ^2^ = 0.98585	*R* ^2^ = 0.98751	*R* ^2^ = 0.99501

### 3.3 Osteoblast/SAIB-based depot interactions

#### 3.3.1 Cytotoxicity of the drug delivery systems toward osteoblastic cells

**FIGURE 4:**
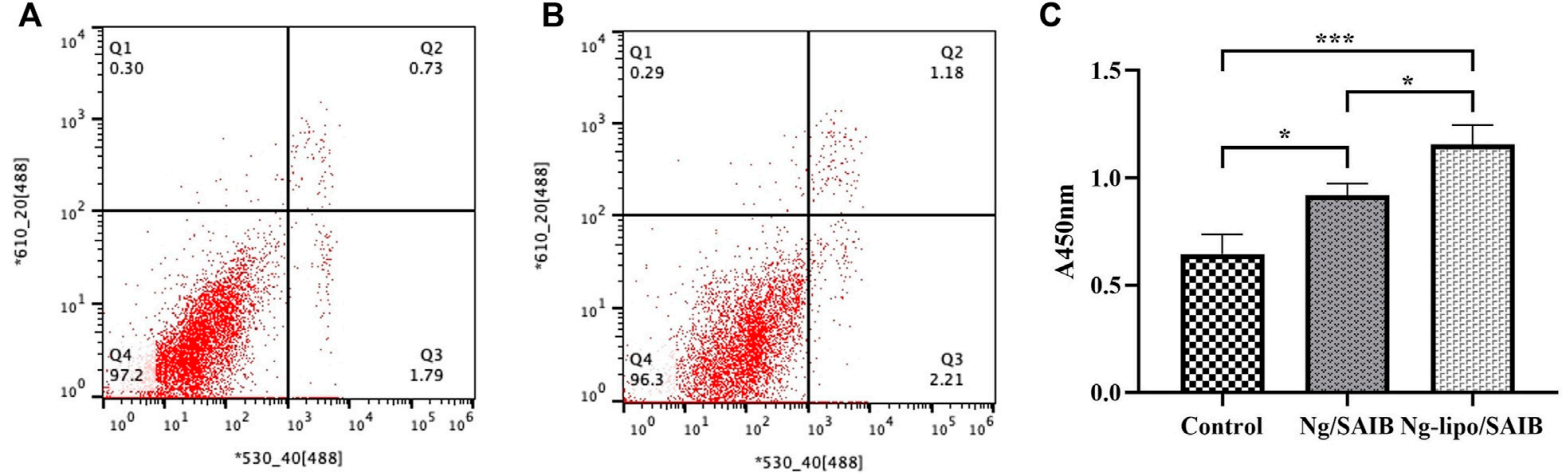
Flow cytometry of apoptotic osteoblast cells assessed using annexin V-FITC/PI fluorescent intensity. Osteoblast cells cultured with basal medium **(A)** and with Ng-lipo/SAIB depot extract solution **(B)**. Cells harvested after 24 h **(C)** CCK-8 assays of cells cultured with basal medium, and extracts of Ng/SAIB and Ng-lipo/SAIB depots (*n* = 3, *p < .05).

As shown in Figures 4A,B, the osteoblast cell survival rate was higher than 90% in the Ng-lipo/SAIB group (96.3%), which was comparable to that of the control group (97.2%). In the CCK-8 assays, osteoblasts cultured with both the Ng/SAIB and Ng-lipo/SAIB extracts showed significant proliferation compared with the blank control (control vs. Ng/SAIB:*p* = .041,control vs. Ng-lipo/SAIB:*p* = .0006). Moreover, Ng-lipo/SAIB promoted the proliferation of osteoblasts better than Ng/SAIB (Figure 4C optical density (OD): .9193 vs. 1.155, *p* = .0276).

**FIGURE 5:**
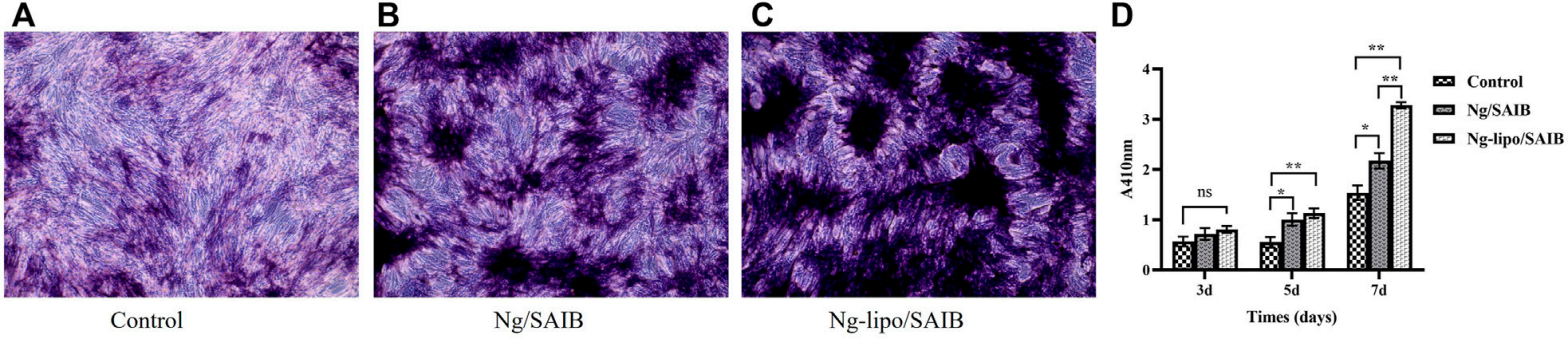
Osteogenic differentiation capacity of osteoblasts cultured with basal medium, and extracts of Ng/SAIB and Ng-lipo/SAIB, respectively. Representative ALP staining at 7 days **(A–C)**. Quantitative ALP activity at 3, 5, and 7 days **(D)**. (*n* = 3, *p < .05, ***p < .005, ****p < .0005).

ALP is an early-stage marker of osteogenesis (Deng et al., 2019). ALP staining at 7 days was more intense for the osteoblasts cultured with the Ng-lipo/SAIB extracts than with the other two groups (Figures 5A–C). Moreover, as shown in Figure 5D, further quantitative analysis showed that the ALP content in the cells cultured with the Ng-lipo/SAIB extracts was significantly higher than that with the control group on the 3rd (OD: .8073 vs. .5687, *p* = .0593), 5th (OD: 1.1310 vs. .5522, *p* = .0052), and 7th days (OD: 3.278 vs. 1.536, *p* = .0012).

### 3.4 *In vivo* study

No suppuration or exposure of the material was observed in the defect sites during 2 and 8 weeks of healing.

#### 3.4.1 Micro-CT assessment of the new bone formation

**FIGURE 6:**
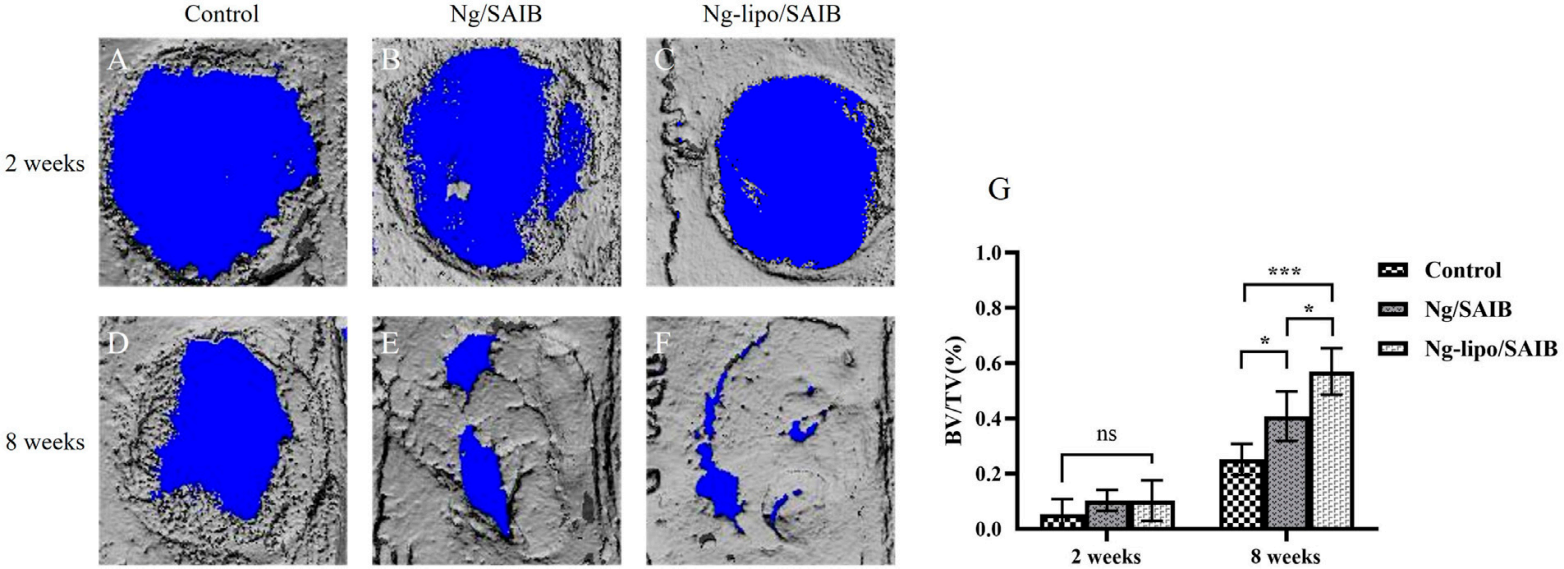
Representative 3D images of reconstruction **(A–F)**. Statistical quantification **(G)** of the new bone formation rate (*n* = 3, *p < .05, ***p < .0005).

Figures 6 A–G presents the representative 3D micro-CT images and the corresponding BV/TV data at 2 weeks and 8 weeks after surgery. No significant difference was observed among the three groups after 2 weeks (*p* > .05). After 8 weeks of bone defect healing, significantly improved bone formation was observed in the Ng-lipo/SAIB group, with the mean BV/TV reaching 57.0%, while the mean BV/TV values of the control and Ng/SAIB groups were 25.18% and 40.80%, respectively (P1 = .0003, P2 = .0331).

#### 3.4.2 Histological and IHC assessment of new bone formation

**FIGURE 7:**
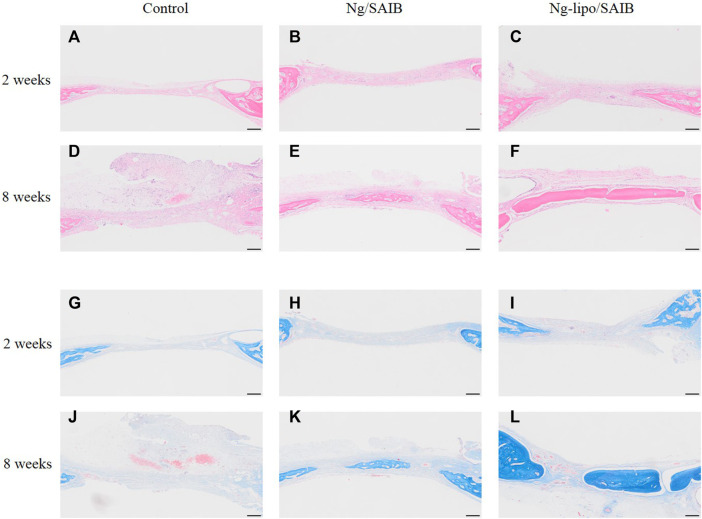
Representative HE **(A–F)** and Masson **(G–L)** staining of the bone regeneration region. Images of different groups at 2- and 8-weeks post-surgery are shown. Scale bar = 200 μm.

The results of HE staining showed that the defect was occupied by connective tissue at 2 weeks after surgery, in which a few inflammatory cells were observed, even in the SAIB-based groups (Figures 7A–F). Under Masson staining, osteoids displayed mostly blue collagenous fibers, which indicated that the newly formed tissue was undergoing active osteogenesis. Moreover, the newly formed tissue in the Ng-lipo/SAIB group was denser and more mature than in the Ng/SAIB group (Figures 7G–L).

**FIGURE 8:**
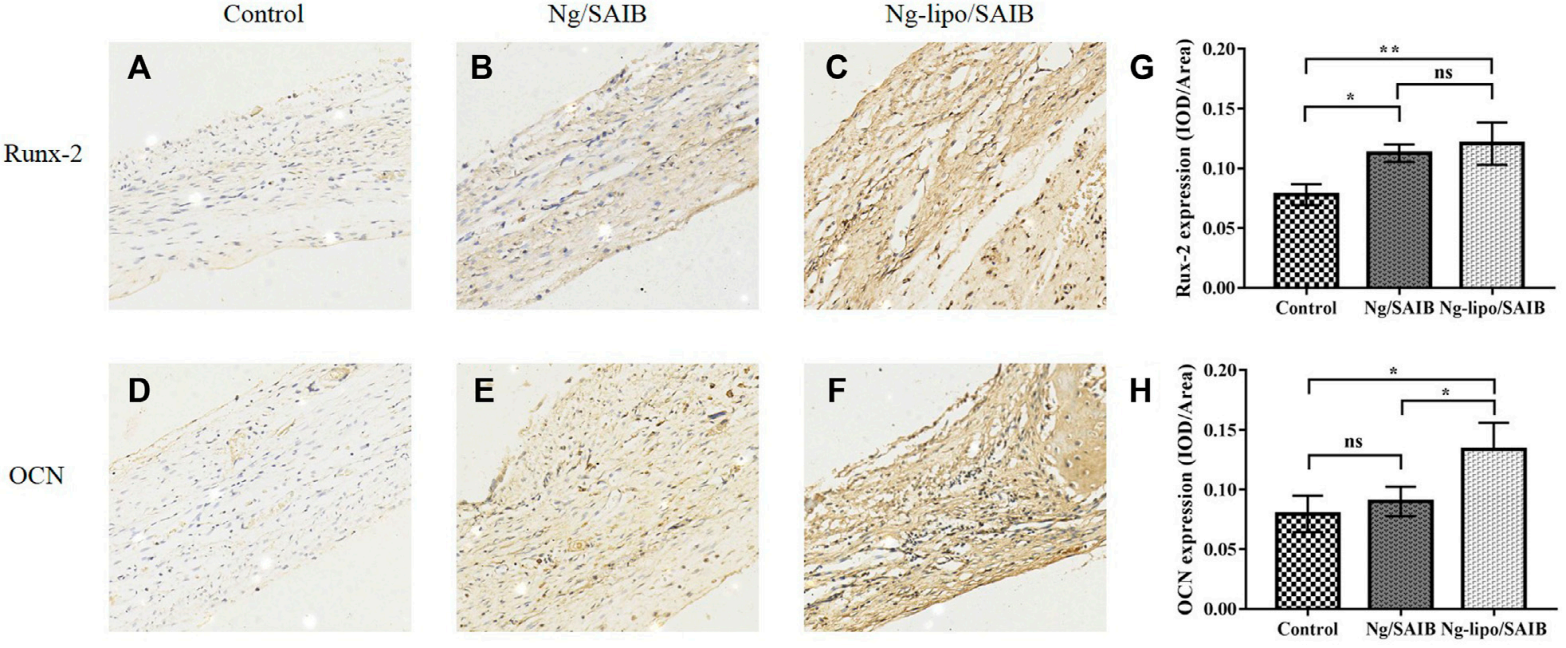
Representative immunohistochemical staining of the osteogenic marker Runx-2 after 2 weeks **(A–C)** and osteogenic marker OCN after 8** **weeks **(D–F)**. The corresponding quantitative comparison of osteogenic expression of Runx-2 after 2** **weeks **(G)** and OCN after 8** **weeks **(H)** (*p < .05, **p < .005, ns, no significance). Scale bar, 20 μm.

Figure 8 shows the results of representative IHC staining for Runx-2 and OCN. Compared with the control and Ng/SAIB groups, the levels of Runx-2 and OCN were significantly higher in the Ng-lipo/SAIB group. This was verified by the quantitative study of osteogenic expression.

## 4 Discussion

This work concentrated on carriers with enhanced capabilities for the prolonged release of Ng, which has demonstrated effectiveness in promoting bone growth (Chen et al., 2016). In this study, freeze-dried Ng liposomes were incorporated with SAIB for the first time. Our results suggested that the Ng-lipo/SAIB depots could reduce drug the burst release in the case of an excessive concentration of Ng, usually resulting in a toxic effect, thus improving the therapeutic outcome of osteogenesis.

The major component of commercial soybean lecithin is phosphatidylcholine (PC). PC did not stimulate more lipid droplet accumulation in osteoblasts (Chang et al., 2018). From this point of view, Phosphatidylcholine-containing liposomes could provide the best liposomal formulation for treating bone diseases. Previous studies reported that cholesterol could increase the hydrophobia of the membranes (Subczynski et al., 1994), which can favor the inclusion of hydrophobic molecules. This explains the gradual increase of EE of liposomes when the cholesterol increased from 0 mol% to 30 mol% in the current study. The liposome formulation of 70:30 (lipid/cholesterol, mol/mol) produced the highest encapsulation rate, which is consistent with the results of previous experiments (Briuglia et al., 2015). Ng is a hydrophobic molecule embedded in phospholipid bilayers, which comprise a competitor with cholesterol (Deniz et al., 2010). Thus, the highest EE indicated that the spatial position of the liposome membrane reached saturation. This could be a potential explanation for the decrease of EE with the continual increase of the cholesterol ratio.

The diameter of freeze-dried Ng liposomes met our requirements (<200 nm), and similar results were obtained by Yang and Kotta (Yang et al., 2012; Kotta et al., 2021). At neutral pH, the negative charge of phosphorus groups is normally counteracted by the positive charge of choline and ethanolamine groups, resulting in the absence of a net charge in PC. However, relatively high levels of anionic phospholipid classes, such as phosphatidylinositol and phosphatidic acid (PA), which have also been reported in soybean lecithin, would result in a negative membrane surface charge (Taladrid et al., 2017). The largely negative zeta potential indicated electrostatic repulsion owing to a significantly negative surface charge responsible for the stability of the liposomes (Lin et al., 2014). Anionic liposomes have biocompatibility and stability fairly, whereas cationic liposomes induce apoptosis in interaction with cells (Aramaki et al., 1999). In addition, the freeze-dried liposomes showed a narrow size distribution because of the low PDI value (<.3) (Danaei et al., 2018). These results suggested that the liposomes were homogeneous.

The combined vibrational absorbance bands of the drug-loaded liposomes and their combination with the SAIB depots were visible in the FTIR spectra, with no significant shifting. The results indicated that their compositions were unaltered.

Experiments on the release of drugs *in vitro* can reflect not only the quality features of formulations, but also their likely *in vivo* performance, which is a necessary step for the development of a drug delivery vehicle. In our *in vitro* study, Ng liposomes alone had a burst release close to 74% and showed no improvement compared to other advanced products, such as encapsulating Ng in metal-organic frameworks into liposomes (Wang et al., 2021). The mechanism of the liposomes’ *in vitro* release seems to be the formation of transient pores in the lipid bilayer (Schroeder et al., 2009). The burst release could be caused by a readily available medication that quickly dissolves from the liposomes’ surface or surface degradation induced by interfacial physical or chemical stress. Raising temperature could increase lipid mobility as well as drug diffusion through lipid barriers. (Manna et al., 2019). However, the burst release of Ng was plainly reduced when the liposomes were loaded into the SAIB depots as a barrier against Ng release. Obviously, SAIB provided a secondary barrier for drugs attached to the surface of the liposome membrane to be released into the medium. Moreover, Esters of SAIB could enhance liposome membrane stability physically to decrease drug release (Zhang et al., 2017). The values of the diffusion coefficient (n) in the Ritger–Peppas equation were less than .43 for the Ng-lipo/SAIB group, indicating that the mechanism of drug release from the liposomal SAIB-based depots was diffusion (Cuomo et al., 2015).

The results of the flow cytometry assay suggested that the Ng-lipo/SAIB depots are safe and non-toxic to osteoblast cells. Meanwhile, the Ng-lipo/SAIB depots also presented better potential to promote the osteogenesis of osteoblast cells than the control or Ng/SAIB alone. In addition, the CCK-8 and ALP activity assays showed that Ng-lipo/SAIB had good biocompatibility and could promote cell proliferation and differentiation. According to a previous study, Un et al. confirmed that liposomes are absorbed *via* endocytosis (Un et al., 2012). Ng might be used by liposomal carrier-mediated technology to promote its intracellular uptake to increase its bioavailability.

During the *in vivo* experiments, all the Ng-lipo/SAIB, Ng/SAIB groups, and control groups displayed similar amounts of bone formation 2 weeks after surgery. After 8 weeks post-surgery, significantly improved bone formation was observed in the Ng-lipo/SAIB group, which suggested that the Ng-lipo/SAIB depots played an important role in promoting osteogenesis. The new bone formation in the Ng-lipo/SAIB group increased by 57.0%, even with less Ng than in our previous study (Yang et al., 2019). This was strong evidence that liposomes/SAIB can improve Ng therapeutic efficacy (Wang et al., 2018).

Runx-2 is an essential factor during the early stage of osteoblast maturation (Shen et al., 2021). OCN plays an important role in the mineralization of osteoblasts, representing an important factor in the regulation of osteoblastic differentiation (Kavukcuoglu et al., 2009). Our previous study showed that Ng-loaded microsphere/SAIB could enhance osteogenic differentiation by promoting the expression of OCN and Runx-2 (Yang et al., 2019). In the current study, the levels of Runx-2 after 2 weeks and those of OCN after 8 weeks in the Ng-lipo/SAIB group were significantly higher than those in control, which was consistent with the above result.

The main limitation of this study is that the SAIB was not involved in the phospholipid bilayer of the liposomes, which needs further exploration (Qin et al., 2018; Das et al., 2014). Moreover, it should be noted that there is no more detailed information about the mechanism of drug release from the SAIB depots, and their distribution and metabolism *in vivo*.

## 5 Conclusion

In conclusion, we successfully incorporated freeze-dried Ng liposomes with SAIB to form a SAIB-based hybrid depot. Our results suggested that the Ng-lipo/SAIB depot is a promising drug delivery vehicle to reduce drug burst release and promote controlled drug release over the long term, thus improving the therapeutic outcome of osteogenesis.

## Data Availability

The original contributions presented in the study are included in the article/Supplementary Material, further inquiries can be directed to the corresponding author.

## Glossary

naringin: Ng
liposome: lipo
sucrose acetate isobutyrate: SAIB
β-Hydroxy β-methylglutaryl CoA: HMG-CoA
bone morphogenetic protein 2: BMP-2
Food and Drug Administration: FDA
alpha-modified eagle’s medium: α-MEM
fetal bovine serum: FBS
drug-encapsulation efficacy: EE
drug-loading efficiency: LE
ultraviolet-visible: UV-Vis
dynamic light scattering: DLS
particle size, polydispersity index: PDI
transmission electron microscopy: TEM
Fourier Transform infrared spectroscopy: FTIR
annexin V-fluorescein isothiocyanate: FITC
propidium iodide: PI
cell counting kit 8: CCK-8
enzyme-linked immunosorbent assay: ELISA
alkaline phosphatase: ALP
optical density: OD
5-bromo-4-chloro-3-indolyl phosphate: BCIP
nitro blue tetrazolium: NBT
micro-computed tomography: micro-CT
ethylenediaminetetraacetic acid: EDTA
hematoxylin and eosin: HE
immunohistochemistry: IHC
osteocalcin: OCN
phosphatidylcholine: PC
phosphatidic acid: PA
